# Case Report: Conservative Non-operative Management of a Neonate With Torted Wandering Spleen

**DOI:** 10.3389/fped.2021.791932

**Published:** 2022-01-28

**Authors:** Maha Bourusly, Mariam Ayed, Zainab Bahzad

**Affiliations:** ^1^Pediatric Hematology Oncology Department, National Bank Kuwait Specialized Children Hospital, Kuwait City, Kuwait; ^2^Neonatal Department, Farwaniya Hospital, Kuwait City, Kuwait; ^3^Pediatric Department, Farwaniya Hospital, Kuwait City, Kuwait

**Keywords:** neonate, spleen, wandering spleen, splenectomy, conservative treatment

## Abstract

**Background:**

The management of wandering spleen (WS) with torsion, a rare pathological condition, is currently unclear. Most patients with this disorder are treated with surgical interventions, such as splenectomy or splenopexy.

**Case Presentation:**

A newborn female presented with low hemoglobin (10.8 mg/L) and high total serum bilirubin (193 μmol/L) at 3 h of life. A palpable mass was observed during her physical examination, and a magnetic resonance imaging scan of the abdomen confirmed the presence of an infarcted WS with torsion. Upon conservative management with oral antibiotic prophylaxis, careful observation, and repeated follow-ups, the infant remained clinically stable. At 2 years of age, she had normal complete blood count, and a repeat technetium study revealed two splenunculi/splenules in the splenic bed.

**Conclusion:**

Most patients with WS are treated surgically with splenectomy or splenopexy. Non-operative management may be a feasible treatment option in select cases with infarcted WS and may allow the natural process of autosplenectomy to occur.

## Introduction

The spleen is a secondary lymphoid organ normally located in the left upper quadrant of the abdominal cavity that plays an important role in immune defense by keeping harmful microorganisms and defective red blood cells out of circulation (1). Wandering spleen (WS), or ectopic spleen, is a rare congenital or acquired clinical entity (2, 3) predominantly found in children aged <10 years and adult females aged 20–40 years (4, 5). WS is characterized by an elongated vascular pedicle and splenic hypermobility caused by the absence or laxity of the suspensory splenic ligaments that attach the spleen to its normal position (6). This translocated or mobile spleen is further predisposed to torsion, often leading to possible life-threatening complications, such as pancreatitis, gastric volvulus, gastric and duodenal compression, splenic infarction, ischemia, pseudocyst, necrosis, and hemorrhage (7–9).

Abdominal ultrasound and computed tomography (CT) are the preferred imaging modes of investigation that can confirm the diagnosis and provide valuable information for the preoperative evaluation of WS (10, 11). Surgery is usually considered the gold standard treatment of WS with splenic torsion (12). Previous studies have described the presentation of children with WS, its management, and outcome (13, 14); however, descriptions of conservative management in the literature are scarce (15).

We report a rare case of a female neonate presenting with a torted WS at birth. She was successfully treated by a conservative non-surgical approach, with an excellent overall outcome. Further medical investigations of the patient at 2 years revealed the presence of an accessory spleen or splenules without any complications.

## Case Presentation and Follow-Up

A full-term female infant was born at 39 weeks gestation to a 36-year-old mother (O blood type; Rh-positive) *via* spontaneous vaginal birth. The antenatal visits and ultrasound scans of the mother were unremarkable, and antenatal serology testing for hepatitis B, rubella, syphilis, and HIV were negative. The neonate cried immediately at birth, and her Apgar scores were normal: 8 at 1 min and 9 at 5 min. Cord complete blood count (CBC) showed low hemoglobin (10.8 mg/L), hematocrit (33.6%), white blood cell count (8.6 × 10^9^), and platelet count (229 × 10^9^). Her blood group was B positive and the direct Coombs test was abnormally positive. At 3 h of age, a repeat investigation showed high total serum bilirubin (TSB; 193 μmol/L). Therefore, the neonate was admitted to the neonatal intensive care unit for further investigation and treatment.

At the time of admission, the infant was active and stable; her heart rate was 140 beats/min, her respiratory rate 40 breaths/min, and she was breathing normally in normal saturated room air. The infant's weight on admission was 2,300 g (3rd−5th percentile), length 48 cm (25th percentile), and head circumference 33 cm (10th percentile). Cardiovascular, respiratory, and nervous system examinations were unremarkable. No dysmorphic features were noted. However, her physical examination revealed scleral icterus and abdominal distension with a palpable mass in the left hypochondrium. The septic screen was negative, and liver and renal function tests were unremarkable.

The patient was treated empirically with antibiotics and placed on maintenance intravenous fluid. Packed red blood transfusion and triple phototherapy were initiated, and she received intravenous immunoglobulin (0.5 g/kg).

**Figure 1:**
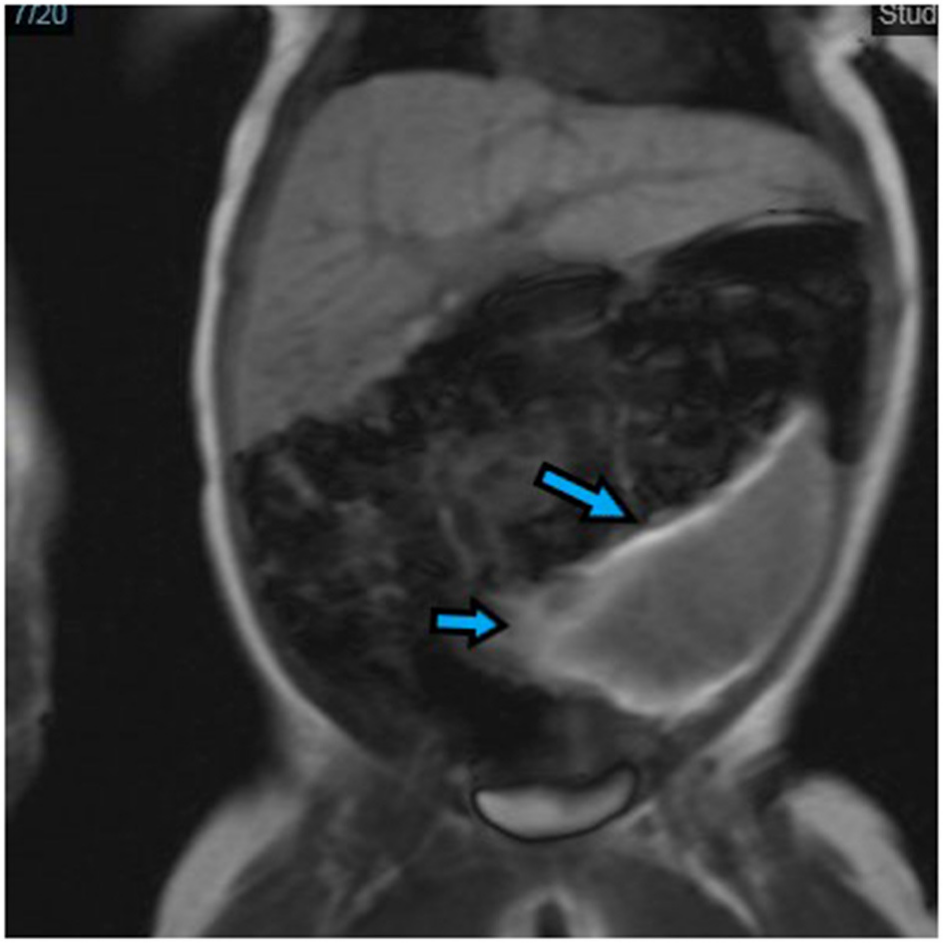
Frontal view of magnetic resonance imaging (MRI) of the abdomen. Wandering spleen (arrow) presented as a well-defined semilunar-shaped structure in the left lumbar region with the descending colon lateral and the left kidney posterior in the lower abdomen. It is seen anterolateral to the small bowel and just beneath the anterior abdominal wall muscle.

An abdominal ultrasound on day 1 revealed moderate ascites and a heterogenous hypoechoic lesion anterolateral to the left kidney that measured 5 × 5.5 × 2.5 cm and extended down to the left iliac fossa, displacing the colon laterally but with no vascularity on Doppler. The mass was intraperitoneal rather than retroperitoneal and suspected to be a hematoma. On day 4, magnetic resonance imaging (MRI) of the abdomen showed a well-defined semilunar-shaped structure in the left lumbar region lateral to the descending colon. The left kidney was posterior to the structure and showed no enhancement after intravenous injection of gadolinium except at its hilum. The picture was indicative of an infarcted WS (Figure 1).

**Figure 2:**
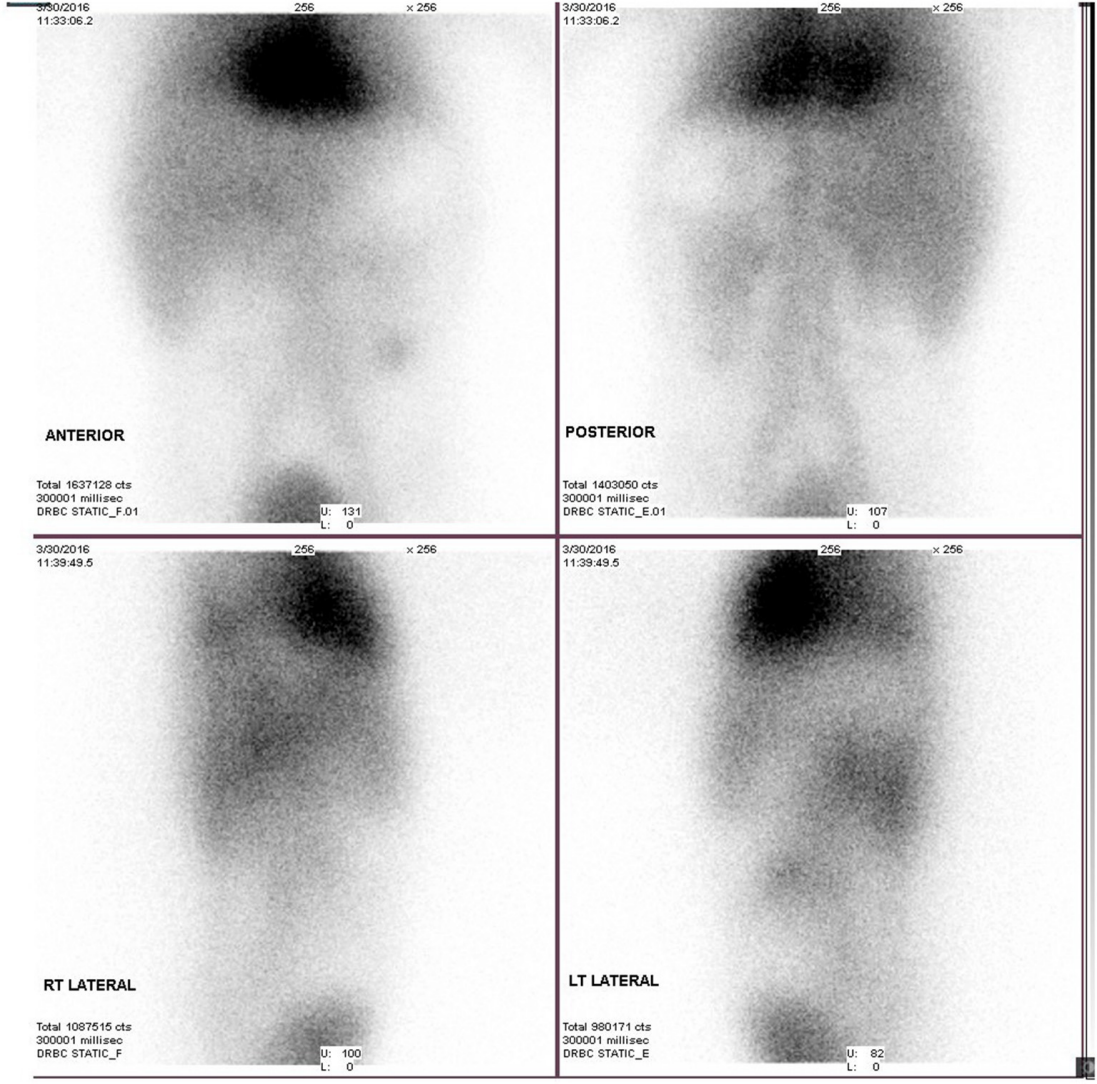
Technetium scan at 5 days old showed absent/non-visualized, non-functional spleen.

A technetium scan at 5 days of life showed an absent/non-visualized, non-functional spleen (Figure 2). Due to the patient's clinical stability, both the medical team and her parents decided to proceed with conservative non-surgical management. The patient was provided supportive care and discharged at 18 days of age with acceptable CBC values. Prophylactic amoxicillin was continued and the child was seen regularly by general pediatrics and pediatric hematology services. A follow-up ultrasound at 3 months of age was consistent with previous ultrasound findings, except that the size of the WS had decreased to 2.7 × 1.2 cm with early calcified foci.

**Figure 3:**
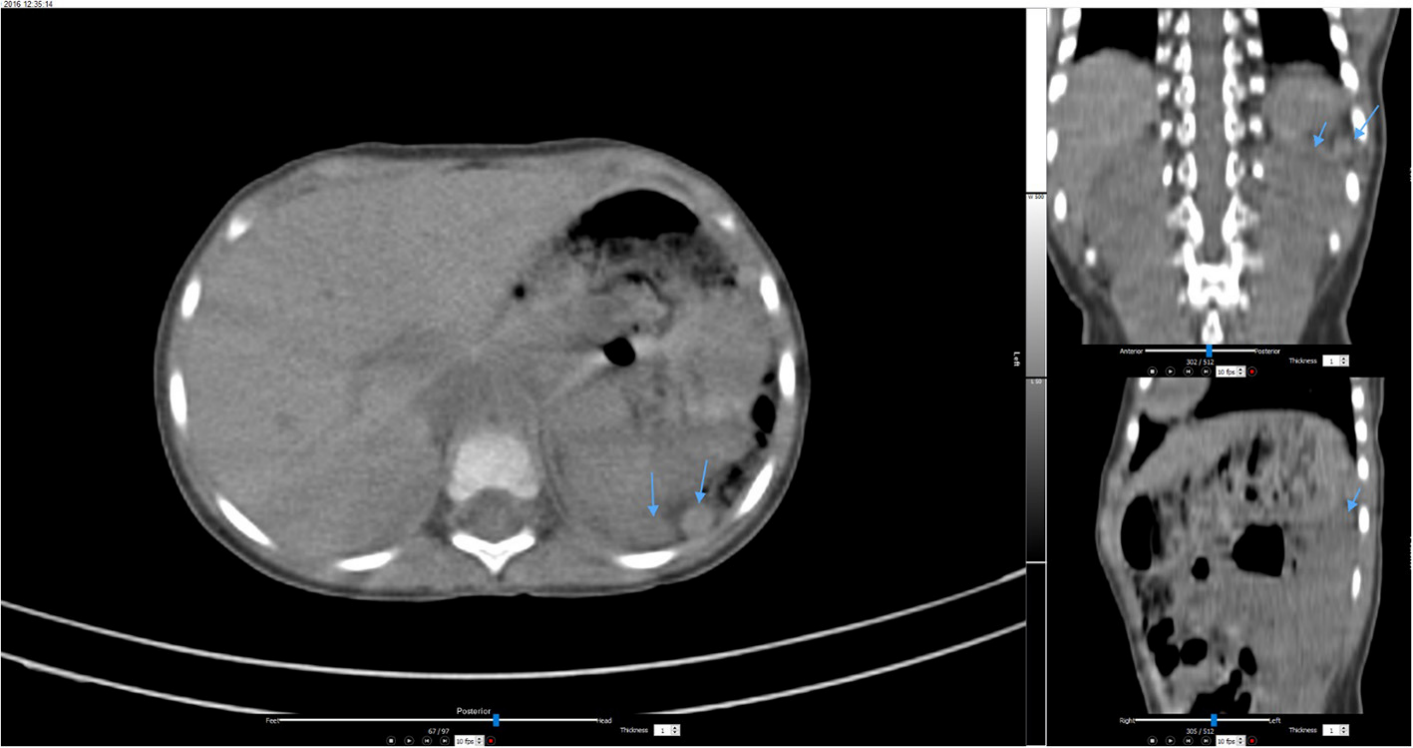
Single-photon emission computed tomography (SPECT-CT) images of the abdomen at 2 years of age showed two oval foci in the left upper abdomen (arrows) indicating splenunculi/splenules in the splenic bed.

**Figure 4:**
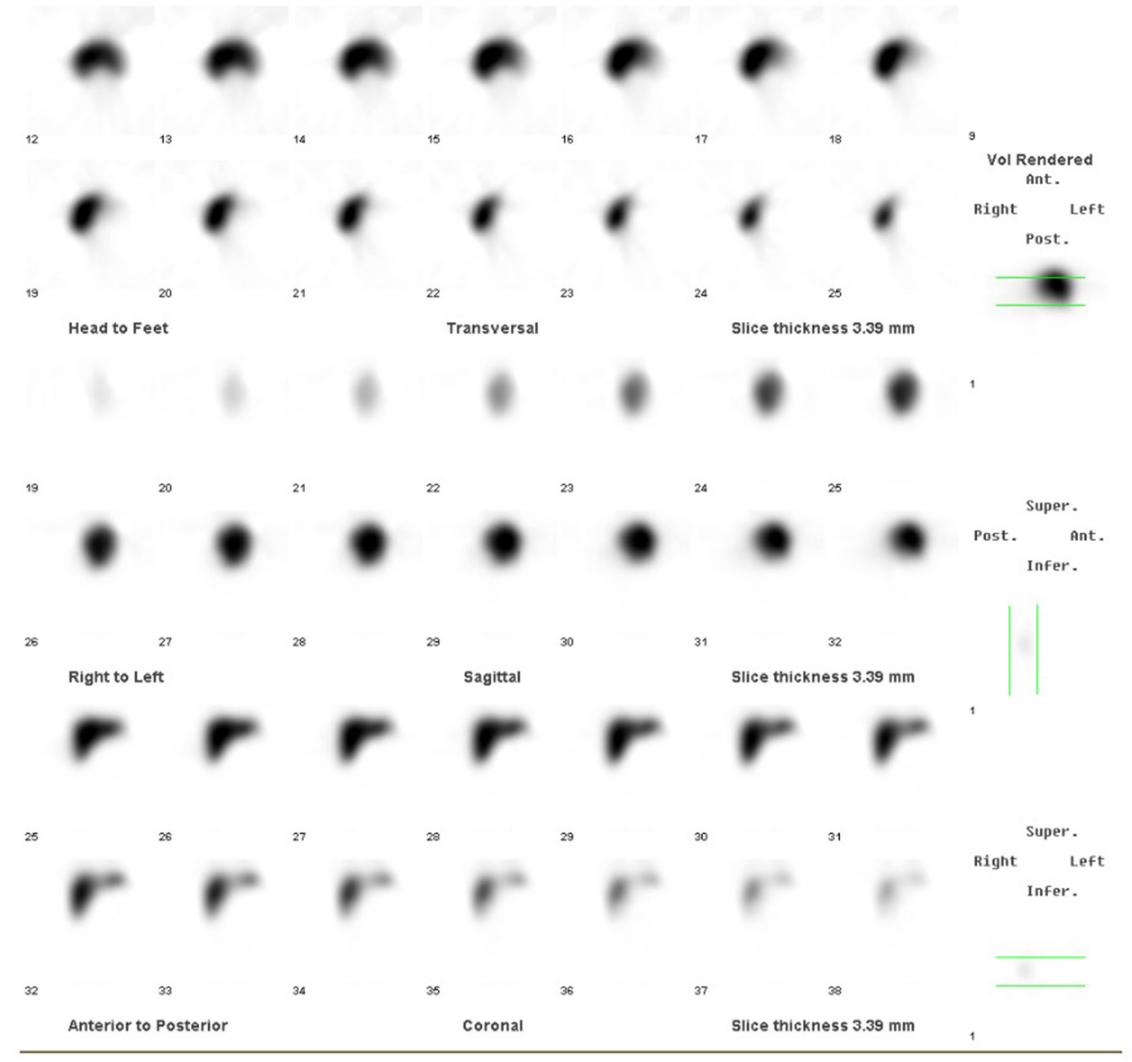
Follow-up technetium scan at 2 years of age showed absent/non-visualized, non-functional spleen.

The child gained weight appropriately during the first 2 years of life. She was admitted once to the general pediatric ward with viral bronchiolitis. Lab investigations revealed mild microcytic hypochromic anemia, leukocytosis, and mild thrombocytosis (600–1,000 × 10^9^). Considering her hemogram, the nuclear study was repeated. Static and single-photon emission CT (SPECT-CT) imaging demonstrated two small oval foci of uptake posteriorly in the left hypochondrium, indicative of splenunculi/splenules (Figure 3). A follow-up technetium scan still showed an absent/non-visualized, non-functional spleen (Figure 4). Findings were consistent with asplenia with splenunculi/splenules in the splenic bed.

A 3-year follow-up showed improved blood indices within normal limits. The parents chose to stop her oral penicillin at 3 years of age. The child continued to thrive normally and was subsequently discharged from the pediatric hematology clinic.

## Discussion

Here, we reported the case of a female newborn who presented with an abdominal mass, hyperbilirubinemia, and anemia. The diagnosis was not clear in the first 4 days of life and was considered to be either hemolytic anemia or postnatal trauma. Further evaluation by MRI confirmed an infarcted WS, and a technetium scan revealed an absent or non-functional spleen.

WS accompanied with torsion is rare in pediatric patients, accounting for <0.2% of all patients undergoing splenectomy (3). In children, the most common causes of WS are congenital maldevelopment and abnormal fixation of ligaments to the overlaying peritoneum of the spleen (3). Acquired WS has been reported to be associated with other conditions, including immunoglobulin deficiency, Gaucher disease, and DiGeorge syndrome (3, 8, 15, 16). Diagnosis of WS in children is often challenging due to either an absence of symptoms, ambiguous clinical presentation, or non-specific symptoms, such as abdominal pain, fever, nausea, and vomiting. If left undiagnosed, splenic torsion may result in splenic infarction and/or thrombosis of the pedicle (8, 17). An infarcted spleen may further undergo hemorrhagic transformation or become systemically infected, leading to a fatal outcome. Therefore, timely diagnosis through Doppler ultrasonography, CT, MRI, scintigraphy, and splenic angiogram is helpful in saving the spleen and the life of the patient (9, 10, 18).

Depending on the viability of the spleen, complete splenectomy, partial resection *via* laparoscopy, or open surgical approaches (*via* laparotomy, splenopexy, and gastropexy) are often recommended for most patients with a WS and infarction, thrombosis, hemorrhage, splenomegaly, or compression of surrounding organs (12, 13, 19, 20). However, despite having a completely infarcted spleen, our patient remained completely stable. With time, her hemoglobin and platelet levels were maintained. Fortunately, she did not develop any childhood infection even though her vaccination schedule was delayed due to the possibility of an ineffective immune system. The patient remained free of infection by maintaining a regimen of antibiotic prophylaxis and regular follow-up visits. She managed to catch up developmentally at 2 years of age.

Watchful waiting or conservative non-operative methods for successfully managing WS with torsion have been described in a few other cases (21–23). Non-operative management of our patient allowed the course of splenic infarction to continue naturally. Follow-up at 2 years revealed asplenia with the development of accessory spleen/splenunculi in the splenic bed. Splenunculi or auto-transplantation of splenic tissue is thought to provide immune protection against sepsis. These patients no longer require antibiotic prophylaxis and are at no greater risk of overwhelming infection than the healthy population (23). Our patient was taken off antibiotics and was doing clinically well at 3 years.

## Conclusion

The excellent clinical outcome of our patient suggests that conservative medical treatment may be a reasonable alternative to unwarranted surgical intervention in select clinically stable pediatric patients with a completely infarcted spleen, avoiding the possible complications of surgery. A careful review of imaging scans, early diagnosis to rule out this uncommon clinical condition, close monitoring, and timely treatment of the patient are important in evading mortality.

## Data Availability Statement

The raw data supporting the conclusions of this article will be made available by the authors, without undue reservation.

## Ethics Statement

The studies involving human participants were reviewed and approved by Ministry of Health of Kuwait and the National Bank Kuwait Specialized Children Hospital, Kuwait. Written informed consent to participate in this study was provided by the participants' legal guardian/next of kin. Written informed consent was obtained from the individual(s), and minor(s)' legal guardian/next of kin, for the publication of any potentially identifiable images, or data included in this article.

## Author Contributions

MB and MA conceptualized and designed the study, drafted the initial manuscript, and reviewed and revised the manuscript. MB and ZB designed the data collection instruments and collected data. All authors approved the final manuscript and agree to be accountable for all aspects of the work.

## Conflict of Interest

The authors declare that the research was conducted in the absence of any commercial or financial relationships that could be construed as a potential conflict of interest.

## Publisher's Note

All claims expressed in this article are solely those of the authors and do not necessarily represent those of their affiliated organizations, or those of the publisher, the editors and the reviewers. Any product that may be evaluated in this article, or claim that may be made by its manufacturer, is not guaranteed or endorsed by the publisher.
